# Announcement: First International Conference on Metals and the Brain

**DOI:** 10.1155/MBD.2000.56

**Published:** 2000

**Authors:** Paolo F. Zatta

**Affiliations:** Center for the study of Physiology and Biochemistry of Metalloproteins University of Padova Department of Biology Via Trieste, 75 Padova 1-35131 Italy


					FIRST
From
INTERNATIONAL CONFERENCE ON
METALS AND THE BRAIN
Neurochemistry to Neurodegeneration
University of Padova, Italy
September 20-23, 2000
Despite the importance of metal ions in several biological processes, there has been, until recently,
little molecular information available on their active role in the brain. The molecular mechanisms of
metal transporters, for instance, is of great importance in that an increasing number of human
diseases are thought to be related to disturbances in metal ion homeostasis (overload or
deficiency) (e.g. Menkes and Wilson's disease, hepatic encephalopathy, uremia etc.) and in some
neurodegenerative diseases (e.g. Alzheimer's and Parkinson's disease, Friedreich's ataxia,
immunodeficiencies and others).
The aim of this conference is to establish the state of art in this important field sharing scientific
knowledges and professional experiences in a strongly interdisciplinary atmosphere together with
the opportunity of creating collaborative networks between clinical sciences and basic research.
Padova is an old historical city, located 25 km from Venice, with a prestigious University established
since 1222, where eminent scientists like Galileo Galilei, Marcello Malpighi, William Harvey, just to
mention a few, built scientific milestones, and where you can still admire the first anatomic theatre
and the oldest Botanical Garden.
Scientific Program
Aging, metal ions accumulation and oxidative stress
Brain markers
Chemistry (speciation and analytical procedures)
Experimental models
Metallothionein isoforms in human and experimental neuropathology
Neurochemistry and Enzymology
Neurotoxicology
Neurodegenerative processes
Hepatic Encephalopathy
Neuropharmacology
Environmental risks
Occupational Medicine
Metal ions deficiency in malnutrition and brain development
Uremia and metal ions dismetabolism
If you plan or wish to attend the conference, in order to be in the mailing list for the second and final
circular, send IMMEDIATELY the participation format that you will find, together with further details,
at
http:www.bio.unipd.it/~metalpro/confer/confer.htm
or mail (fax/e-mail) to
Paolo F. Zatta
Center for the study of Physiology and Biochemistry of Metalloproteins
University of Padova
Department of Biology
Via Trieste, 75
1-35131 Padova, Italy
<Zatta @ cribil .bio.u nipd.it>
56

				

